# Orientational order of liquids and glasses *via* fluctuation diffraction

**DOI:** 10.1107/S2052252516016730

**Published:** 2017-01-01

**Authors:** Andrew V. Martin

**Affiliations:** a ARC Centre of Excellence for Advanced Molecular Imaging, School of Physics, University of Melbourne, Parkville, Victoria 3010, Australia

**Keywords:** correlated fluctuations, dynamical studies, XFEL, framework-structured solids and amorphous materials, structure prediction, coherent diffraction

## Abstract

Liquids, glasses and other amorphous matter lack long-range order, which makes them notoriously difficult to study. Local atomic order is partially revealed by measuring the distribution of pairwise atomic distances, but this measurement is insensitive to orientational order and unable to provide a complete picture of diverse amorphous phenomena, such as supercooling and the glass transition. Fluctuation scattering with electrons and X-rays is able provide this orientational sensitivity, but it is difficult to obtain clear structural interpretations of fluctuation data. Here we show that the interpretation of fluctuation diffraction data can be simplified by converting it into a real-space angular distribution function. We calculate this function from simulated diffraction of amorphous nickel, generated with a classical molecular dynamics simulation of the quenching of a high temperature liquid state. We compare the results of the amorphous case to the initial liquid state and to the ideal f.c.c. lattice structure of nickel. We show that the extracted angular distributions are rich in information about orientational order and bond angles. The diffraction fluctuations are potentially measurable with electron sources and also with the brightest X-ray sources, like X-ray free-electron lasers.

## Abstract 2

We present a new method of extracting distributions of orientational order, like bond angles, from fluctuation diffraction data. It has potential applications to study of liquids, glasses and other systems that lack long-range order.

## 1. Introduction   

Phases of matter that lack long-range order, such as liquids, glasses and other amorphous phases, derive their physical properties from short-range (<5 Å) and medium-range order (5–20 Å) (Elliott, 1991 ▸). However, at these length scales there is a daunting amount of structural variability and we are far from a full understanding of the macroscopic phenomena that arise from local order like the glass transition (Mauro, 2014 ▸). While short-range order is often a direct product of chemical bonds, like the well coordinated polyhedra of oxide glasses (Elliott, 1991 ▸), the medium-range order that is prevalent in chalcogenides (Salmon *et al.*, 2005 ▸) and metallic glasses (Sheng *et al.*, 2006 ▸; Wu *et al.*, 2015 ▸) is far more complex. Medium-range order is key to the optical, chemical and thermodynamic properties of glass-forming materials (Mauro, 2014 ▸) and the possibility of engineering them (Martin *et al.*, 2002 ▸; Mauro, 2014 ▸), which has made understanding topological order and its extraordinary diversity one of the outstanding challenges in condensed matter and materials science.

Studies of amorphous phases rely heavily on measurements of the pair-distribution function (PDF) (Elliott, 1983 ▸; Fischer *et al.*, 2006 ▸), which is related to the probability of finding an atom at a distance *r* from a given reference atom. The popularity of the PDF is due largely to its accessibility *via* X-ray, neutron or electron diffraction. It is used to validate structural models like molecular dynamics (MD) models (Car & Parrinello, 1985 ▸) and as an input to reverse Monte-Carlo techniques (Fischer *et al.*, 2006 ▸). However, the pair distribution lacks information about orientational order and is frequently insufficient to experimentally measure hidden topological order predicted by MD simulations (Wu *et al.*, 2015 ▸). The deficiency of the PDF is partially addressed by complimentary techniques like nuclear magnetic resonance and Raman scattering (Elliott, 1983 ▸) that can be matched to structural models to infer information about short-range order, like bond angles. Further insight can also be gained by measuring the PDF with elemental specificity with anomalous X-ray diffraction (Elliott, 1983 ▸) or with neutrons *via* isotropic substitution (Fischer *et al.*, 2006 ▸).

Promisingly, fluctuation electron microscopy is developing into a powerful probe of medium-range order (Treacy *et al.*, 2005 ▸). It was used to find nanometre-sized polycrystalline regions in amorphous silicon that were previously undetected in PDF measurements (Treacy & Borisenko, 2012 ▸). Fluctuation diffraction microscopy has also been developed for X-rays at 100 nm resolution (Fan *et al.*, 2005 ▸) and it has been shown that coherent X-ray diffraction is sensitive to local rotational symmetries in disordered materials (Wochner *et al.*, 2009 ▸). Femtosecond X-ray free-electron lasers have been used to study supercooled water (Sellberg *et al.*, 2014 ▸) and could be extended to orientational order if X-ray fluctuation diffraction can be pushed to atomic resolution. However, fluctuation measurements typically take the form of statistical or correlation measures of scattered intensity that, aside from rotational symmetries, are difficult to interpret structurally. Detailed numerical forward models of the structure and the diffraction are usually required (Treacy *et al.*, 2005 ▸). This stands in stark contrast to the PDF which has a direct, invertible relationship to the mean diffraction signal. A similar invertible relationship to real-space statistical distributions has been lacking for fluctuation diffraction measurements.

Alongside the development of fluctuation microscopy, diffraction fluctuations have been proposed as a route to the structures of biological molecules, like proteins, without the need for crystallization (Kam, 1977 ▸). The idea is to measure the diffraction from multiple identical copies of the molecule in liquid suspension and perform a correlation analysis. It can be shown that information about the orientations and separations of individual molecules is lost in the analysis, leaving information that depends only on the internal atomic structure of the molecule. For some time this research was hampered by an inability to measure proteins faster than their rotational diffusion, thereby washing out the diffraction fluctuations and this was solved by freezing samples (Kam *et al.*, 1981 ▸). Nevertheless, it did not become an established technique with synchrotron sources, most likely because the beam intensity required for sufficient signal-to-noise exceeded radiation dose limits. However, there has been an exciting resurgence within the X-ray free-electron laser community (Saldin *et al.*, 2009 ▸; Starodub *et al.*, 2012 ▸) because femtosecond pulses can take snapshot measurements effectively freezing the molecules in place and outrunning radiation damage processes. This allows potential applications to proteins in solution at room temperature and liquid samples that have rapid decoherence times. Structure determination methods were developed at first for molecules with rotational symmetries (Saldin *et al.*, 2011 ▸; Starodub *et al.*, 2012 ▸) and more recently for an arbitrary three-dimensional structure (Donatelli *et al.*, 2015 ▸).

**Figure 1:**
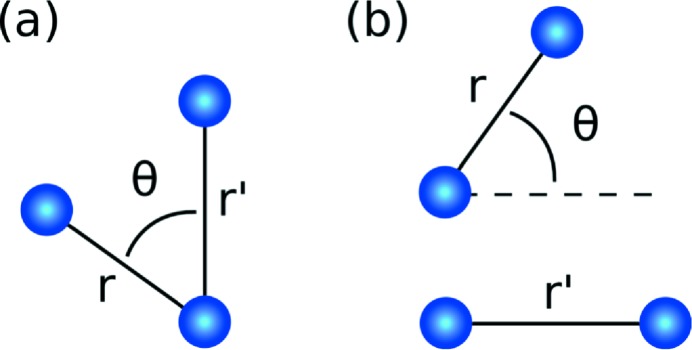
Atom combinations that contribute to the real-space correlation function 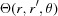 with three atoms (*a*) and four atoms (*b*). The four-atom contributions are insensitive to the separation between the two pairs of atoms.

Underlying fluctuation diffraction methods for biomolecules is a well developed theoretical analysis of intensity correlations in a spherical geometry. Our work here is to show that the same theory can be reapplied and extended in the context of amorphous systems (*e.g.* liquids and glasses) to make fluctuation diffraction data easier to interpret. Here we show how the fluctuations in kinematic far-field diffraction can be mapped into a three- and four-atom correlation function 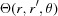 that depends on two pairwise distances and one relative angle (as illustrated in Fig. 1 ▸). It is given by 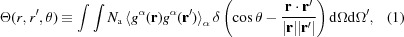
where the average 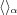 is taken over the ensemble of possible sample configurations, 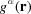 is a three-dimensional two-atom correlation function for a particular sample state α, and 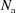 is the mean number of atoms illuminated per measurement. 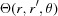 is sensitive to orientation in short- and medium-range order that is absent in the PDF and at small values of *r* and 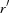 the angular dependence of 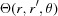 is determined by bond angles.


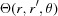
 is also related to higher order correlation functions from statistical physics as follows, 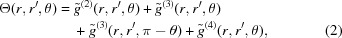
where 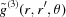 and 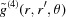 can be derived (see Appendix A) from the respective *n*-body correlation function 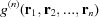 by integrating out the degrees of freedom that the measurement is insensitive to, such as the absolute position of each atomic pair and the absolute orientation of the sample. They are given by 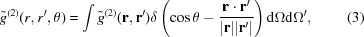


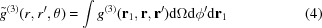
and 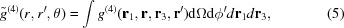
where 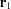 and 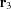 are coordinates of the reference atoms in each pair that are integrated out, and Ω and 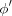 are angular coordinates that specify the absolute orientation of the three- or four-atom group. The *n*-body correlation function 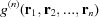 can be described by an average over atomic configurations using delta functions: 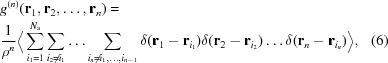
where 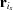 is the position vector for atom 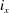, 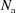 is the number of atoms contained in a sample volume *V*, 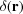 is the delta function and ρ is the number density (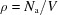). The tilde symbol over the two-body term 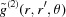 is to indicate that this is not equivalent to the PDF (see Appendix A[Sec appa]), but it is effectively one-dimensional because it is only non-zero when 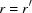 and 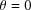 or π.

The function 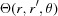 contains information about orientational order through the angular dependence θ, which is related to internal angles in three- and four-atom correlations as shown in Fig. 1 ▸. We use the term orientational order here to refer to non-uniform angular structure in 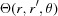. This usage is analogous to the association between the term ‘local order’ and the presence of peaks in the PDF. We thus use the term ‘orientational order’ in broader sense than the term ‘bond orientational order’ (Steinhardt *et al.*, 1983 ▸), which was defined with respect to specific angular metrics for quantifying local structure. The relationship between 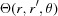 and bond orientational order is of interest for future study.

We note that the theory of local rotational symmetries present in the fluctuation X-ray diffraction of disordered systems developed by Altarelli *et al.* (2010 ▸) overlaps with the work presented here. The key difference is that they conduct the majority of their analysis in Fourier space and focus on symmetry, whereas here we have identified a transformation to real space that makes a direct connection to statistical physics. The connection with statistical physics has been made in the theory of fluctuation electron microscopy (Gibson *et al.*, 2000 ▸) involving correlation functions of the same order as in equation (2). There a forward model of diffraction is presented that is specific to their experimental geometry and they did not identify the inverse relationship that we present here.

## 2. Methods   

### 2.1. Derivation of Θ(*r*, *r*′, θ)   


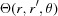
 is obtained from fluctuation diffraction by first calculating the angular correlations of diffraction patterns averaged over an ensemble of different states of the sample. We assume the sample can take an atomic configuration α from a statistical ensemble of possible configurations. We follow the practice developed for pair-distribution analysis (Fischer *et al.*, 2006 ▸) and rescale the diffracted intensity to isolate the structural information. It is convenient to work with 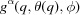 [the Fourier transform of 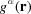], which is obtained by rescaling the kinematic diffracted intensity
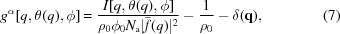
where 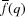 is the mean atomic scattering factor, 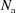 is the number of atoms in the beam, 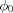 depends on experimental parameters (see Appendix B[Sec appb]) and 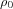 is the mean number density of the sample. We assume these parameters are known. The coordinate *q* is the magnitude of the scattering vector, ϕ is an azimuthal angle around the beam axis and 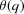 is a polar angle with respect to the beam axis (Saldin *et al.*, 2009 ▸). The last term, 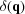, represents low-angle scattering from the mean density which in practice is not measured, but which we include here for completeness. The function 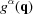 is the Fourier transform of a three-dimensional pair correlation function 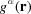 for the sample in state α.

We construct an angular cross-correlation in a similar fashion to existing fluctuation diffraction methods (Kam, 1977 ▸; Wochner *et al.*, 2009 ▸; Saldin *et al.*, 2009 ▸) and take the average over 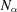 measurements of the sample in different structural states α: 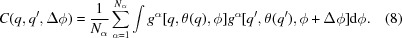
where α is summed over the number of measurements (and, therefore, sample states) and 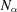 is the number of samples that are measured.

Here we make a key assumption that there is no correlation between the orientation of the sample state and the beam axis. The average of 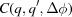 over an ensemble of sample configurations leads to the loss of information about absolute orientation as the number of measurements 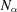 increases. This assumption lets us use a powerful result from the fluctuation diffraction theory for biomolecules that establishes a relationship between 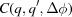 and a series of mutual intensity matrices in a spherical harmonic representation (Saldin *et al.*, 2009 ▸) as follows 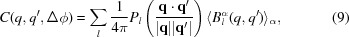
where 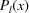 is a Legendre polynomial, 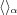 represents an ensemble average and 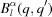 is related to a spherical harmonic expansion coefficients 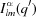 of 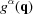 [the Fourier transform of 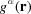] by 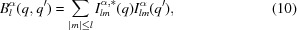
and the spherical harmonic expansion of 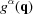 is written 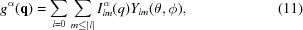
where 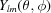 is a spherical harmonic function. Equation (9) is a linear system of equations that can be inverted with standard methods to obtain 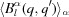, as is done in solution-based biomolecule scattering methods (Saldin *et al.*, 2009 ▸).

In the fluctuation diffraction theory for biomolecules (Kam, 1977 ▸; Saldin *et al.*, 2009 ▸), multiple molecules with identical structure are illuminated and the spherical harmonic expansion defined for the diffracted intensity of a single molecule. In that context, the extracted matrices 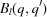 are used to recover a real-space image of the particle or molecule *via* phase retrieval, either by first recovering a three-dimensional Fourier intensity (Saldin *et al.*, 2009 ▸) or by using them directly as constraints for the image recovery algorithm (Donatelli *et al.*, 2015 ▸). In the context of disordered phases of matter, however, every measured state of the sample has a different atomic structure and an imaging analysis is not appropriate. Instead, we diverge from the biomolecular fluctuation diffraction methods by converting the mutual intensity matrices into 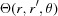 *via* a series of linear transformations and thereby extracting statistical information about short- and medium-range order.

First we use a spherical Bessel transform to map 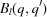 into a function of two real space variables *r* and 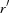. We write the transform as an operator 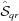 that is given by 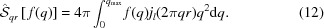
Applying the transform twice, we obtain real-space matrices given by 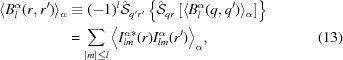
where 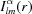 are the spherical harmonic coefficients of 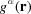. Writing out 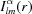 explicitly as a projection of 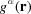 onto the spherical harmonic basis 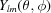, we can evaluate 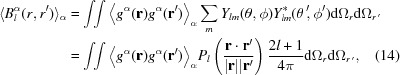
where we have used the following relation to derive the second line 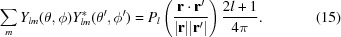
We can construct the following weighted sum to recover 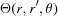: 
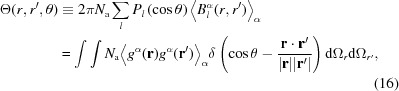
where we have used a known relation for Legendre polynomials: 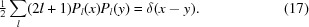
The inclusion of the 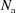 term in equation (16) corrects for the fact that diffraction fluctuations scale as *N*_a_^1/2^, not 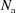, and makes 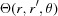 independent of the number of atoms in the sample.

In practice, to recover 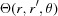 from diffraction measurements 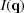, we need to perform the following steps: (i) calculate the angular correlation of each diffraction pattern and average them [equation (8)]; (ii) invert a system of linear equations to recover 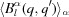 [equation (9)]; (iii) map 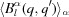 into real space by numerically applying the spherical Bessel transform for both *q* and 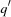 variables at each value of *l* [equation (13)]; and (iv) sum the resulting real-space functions weighted by the Legendre polynomials [equation (16)].

It turns out that ignoring the 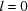 contribution to equation (16), produces 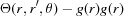, which provides a clearer representation of the angular information as shown in Fig. 3 ▸. As a by-product of ignoring 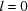 we do not need to explicitly subtract the 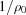 term when rescaling the intensity using equation (7).

### 2.2. Angular symmetries of Θ(*r*, *r*′, θ)   


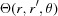
 has angular symmetries which make it unique only in the range 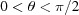. Averaging over absolute orientation has the effect of making 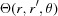 an even function of θ, *i.e.* 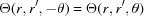. Mathematically, this is because angular information is captured by 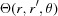 *via* a dot product between the displacement vectors between atomic pairs, 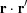, which is proportional to 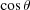, which is an even function of θ.

A second angular symmetry arises because 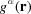 is centrosymmetric, *i.e.* 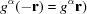. We can write 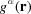 in terms of displacement vectors 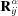 between atoms *i* and *j* in sample configuration α, 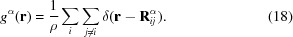
In the above sum, every pair of non-identical atoms contributes two terms with displacement vectors 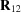 and 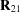, which leads to centrosymmetry. A consequence of this symmetry is that 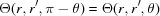.

## 3. Simulations   

### 3.1. Simulation of the extraction of Θ(*r*, *r*′, θ)   

We extracted 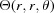 from simulated kinematic diffraction data from nickel in amorphous, liquid and crystalline states. Amorphous nickel is a metallic glass with known medium-range order that can be simulated with classical MD simulations by rapidly quenching from a liquid state. As a by-product, we also generate high-temperature liquid states (3000 K).

**Figure 2:**
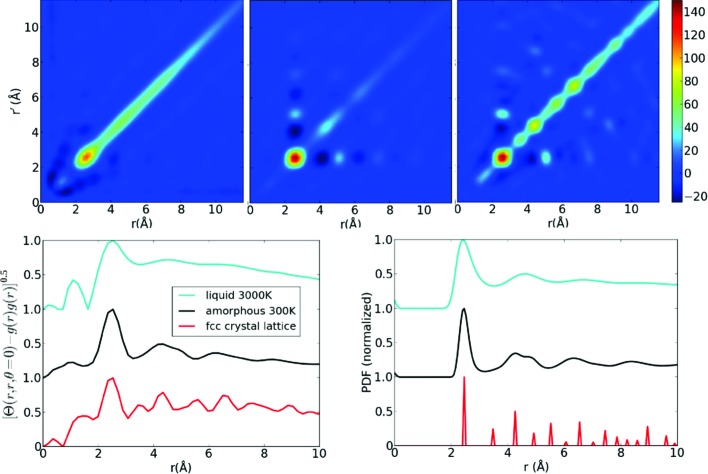
When evaluated at 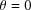, 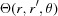 provides information about radial correlations. The top row shows the 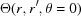 extracted from diffraction simulations of liquid Ni at 3000 K (left), amorphous Ni at 300 K (middle) and an f.c.c. Ni crystal (right). On the bottom left we plot the 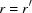 diagonal which contains peak structure that is similar qualitatively to the pair distribution function (bottom right). All line plots have been normalized to have a maximum value of 1 to aid comparison. The information less than 2.5 Å is a numerical artifact related to the finite resolution of the simulation.

**Figure 3:**
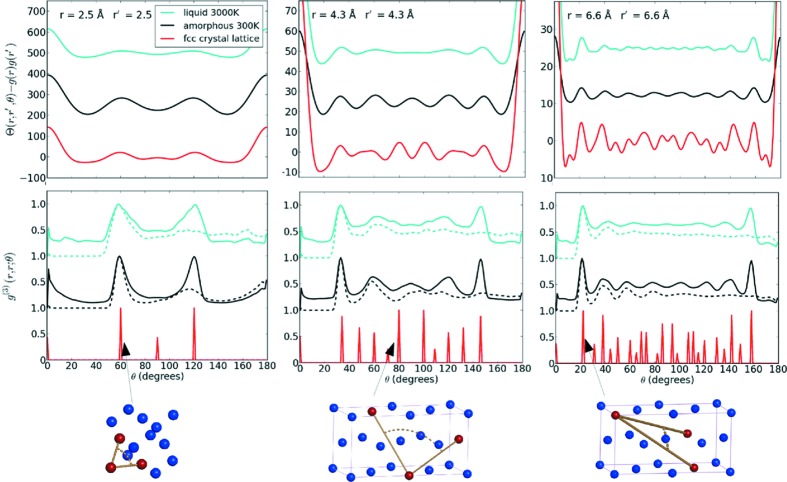
The angular dependence of 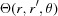 for three different radial shells: 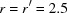
 Å (top left), 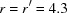
 Å (top middle) and 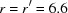
 Å (top right). The peak positions show good agreement with the sum 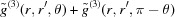 shown by the solid lines in the second row that are calculated directly from the simulated atomic structures. The dashed lines in the second row show the asymmetric correlation 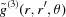 for reference. All 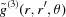 plots have been normalized to a maximum value of unity. The lattice diagrams are examples of the atoms from the f.c.c. lattice (in red) that contribute to peaks in 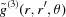. To aid comparison, the plots have been offset and 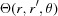 has been scaled by a factor of 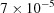 for the crystalline case and by factor of *5* for the liquid case.

The MD simulations were performed with the *LAMMPS* package (Plimpton, 1995 ▸), using the embedded-atom potential and the parameters from Lu & Szpunar (1997 ▸). The supercell size was 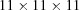 f.c.c. unit cells with lattice parameter 3.52 Å, which contains 5324 atoms. Atoms were randomly displaced from equilibrium positions up to a maximum distance of 0.17 Å, then the system was equilibrated at 3000 K for 40000 time steps using the isothermal-isobaric ensemble (NPT). Each time step was 2.5 fs. The system was then cooled at a rate of 248 K ps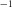 to 300 K and then run for a further 40 000 time steps at 300 K. One thousand different amorphous configurations were generated by the same procedure. The liquid states were recorded after the first 40 000 time steps at 3000 K. The PDFs generated by the simulation are shown in Fig. 2 ▸ (bottom right) and the bond angle distribution is given by the dashed lines for the distance 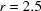 Å shown in Fig. 3 ▸ (bottom left). Coordination statistics for the amorphous state are: 25.7% 12-fold coordinated, 52.5% 13-fold coordinated and 17.8% 14-fold coordinated. These were calculated including neighbours within the first minimum of the PDF located at 3 Å.

For a simple illustration of the principle, we have used small sample volumes (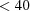 Å) and not modelled noise on the diffraction patterns. The experimental implications of noise and larger sample volumes are discussed in Section 4.3.

X-ray kinematic diffraction simulations were performed for each liquid and amorphous sample configuration at a wavelength of 0.5 Å and a maximum scattering amplitude of 1.38 Å^−1^. Atomic scattering factors were taken from Waasmaier & Kirfel (1995 ▸). Atoms within a 40 Å diameter sphere were used in the diffraction calculation, ensuring adequate sampling on a 128 × 128 *q*-space grid. This was purely for computational convenience, as larger sample volumes and finer grid sizes do not change the final result, but are slower to calculate and require averaging over more configurations to converge. No absorption was modelled.

The inversion of equation (9) was performed with singular value decomposition (SVD) using a maximum *l* value of 40. The elements of the matrix to be inverted are given by 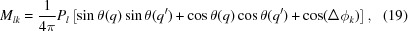
where 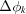 is a discrete sample of the internal angle between two pixel coordinates 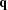 and 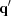, and 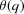 is a polar angle that depends on the magnitude of a pixel coordinate vector [see equation (9) for further details on the geometry]. A separate matrix inversion is thus required for each value of *q* and 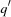. We use 402 angular sampling points (*k* points). Friedel symmetry was applied by setting 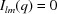 for odd values of *l*, and excluding these from the SVD analysis greatly improved numerical stability. The condition number of the inversion is around 10 when 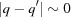, and worsens as 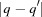 increases. A cutoff was applied to exclude all singular values below 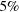 of the largest singular value. The discrete spherical Bessel transform (DSBT) (Lanusse *et al.*, 2012 ▸) was used to map the 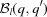 matrices to real space [equation (13)]. The DSBT has a boundary condition that requires 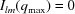. In our simulations, 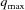 is set to the value measured at the edge of the detector and a Gaussian filter was applied to each diffraction pattern with a width of 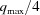 to ensure the boundary condition was met, thereby minimizing numerical errors for a reduction in the effective resolution by a factor of 4. The reduced resolution is likely to contribute to the unphysical non-zero correlation below 2.5 Å shown in Fig. 2 ▸.

The 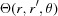 extracted from our simulation displays a peak structure that is dominated by two- and three-atom correlations. Two-atom correlations are prominent along the line 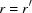 and 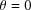 as shown in Fig. 2 ▸, which displays peaks in the same positions as the pair-distribution function for all three phases of the sample. Three-atom correlations appear when we plot the 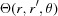 as a function of θ, revealing the orientational order. The majority of angular peaks shown in the top row of Fig. 3 ▸ correspond well to 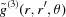 calculated directly from the atomic structures, as shown in the bottom row of Fig. 3 ▸.

## 4. Prospects for experiment   

### 4.1. Electrons   

Measurements of 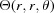 should be possible *via* fluctuation electron diffraction. Cross-correlation functions similar to equation (8) have already been measured with electrons (Gibson *et al.*, 2010 ▸) and could be converted into a measurement of 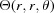 using the methods described here. For electrons, multiple scattering will place limits on sample thickness of around 100–200 nm and further work is needed to establish these limits more precisely. Effects of multiple scattering can be corrected in PDF analysis (Anstis *et al.*, 1988 ▸) and it may be possible to extend these techniques to diffraction fluctuation measurements. Beam profile effects are another known issue (Gibson *et al.*, 2000 ▸) that are important if there is non-uniform illumination on length scales below the correlation length of the sample. A further issue is induced sample dynamics that have been invoked to account for the observation of lower than expected contrast in electron intensity correlation measurements (Rezikyan *et al.*, 2015 ▸).

### 4.2. X-rays   

An advantage for X-rays over electrons is that the kinematic diffraction approximation has greater validity. However, it is more difficult to focus X-ray beams and diffraction fluctuations diminish unfavourably relative to the total scattering as the number of illuminated atoms increases. The best nanofocus X-ray beams are 15 nm or better in diameter, but more commonly they are greater than *25* nm (Sakdinawat & Attwood, 2010 ▸), which is at least an order of magnitude larger than the length scales of short- and medium-range order in amorphous matter. Since shot noise increases with the total scattering with the illuminated volume, we would expect that larger illuminated sample volumes require more diffraction measurements for convergence. Shot noise is investigated in detail in the next section.

It is known from fluctuation diffraction of biomolecules that the contribution of random correlations of the diffraction from different molecules produce a noise in the intensity correlation that scales linearly with the number of molecules. As the signal from the autocorrelation of diffraction from each molecule also scales linearly, the ratio of signal to this source of noise is independent of the number of molecules and bigger than one. An equivalent result is expected to hold for amorphous materials with respect to the number of atoms. Hence, we anticipate that noise from uncorrelated atoms will not be as important as shot noise for the feasibility of fluctuation X-ray experiments of amorphous materials.

One key difference between measuring amorphous materials and biomolecules is that biomolecules are typically delivered to the beam in a liquid environment, which generates background scattering that increases noise. It has been estimated to overcome background scattering that around 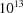 photons per pulse would be required to image protein structures (Kirian *et al.*, 2011 ▸; Kirian, 2012 ▸), which is just beyond the reach of current XFEL facilities (Emma *et al.*, 2010 ▸). However, this issue is not present if we are studying the liquid itself or an amorphous solid, which can be placed in an X-ray beam effectively in isolation from other scattering material. In both experiments there are stray background signals from the X-ray beamline, but methods to reduce these to single photon level are under development for single molecule imaging (Aquila *et al.*, 2015 ▸), which if necessary could be used to for fluctuation diffraction measurements.

These measurements do not require greater beam coherence than typical small-angle X-ray scattering measurements, because the structural correlation length of the sample does not typically extend beyond 2 nm. Although high beam coherence has been a feature of fluctuation X-ray measurements at 100 nm length scales, it becomes less critical as the structural features under investigation become smaller.

### 4.3. Investigation of the effects of noise   

We have investigated the number of patterns required with a statistical model, which is based on an observation from simulation that the diffraction fluctuations scale with *N*_a_^1/2^.

This simulation and the statistical model are detailed in Appendix B[Sec appb]. We can use these statistical properties of the diffraction to derive the number of diffraction patterns required to measure the diffraction fluctuations as a function of experimental parameters. It turns out that the required number of patterns is independent of 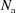, because both the diffraction fluctuations and shot noise have the same dependence on this parameter. The required number of patterns is sensitive to flux and therefore to the focal spot size. In fact, we found that the number of patterns 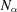 required to measure the diffracted intensity correlation has the following relationship to the beam area *A*, the number of incident photons 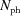 and the mean atomic scattering factor 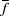: 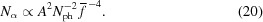
The quadratic and quartic powers indicate that small changes in beam parameters or sample composition can have a big impact on the required number of patterns and place practical limits on the accuracy of the measurement. It will be more challenging to measure lighter elements as 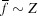. High repetition rate X-ray sources (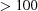 Hz) like X-ray lasers can produce of the order of 10^7^–10^8^ measurements in a 24 h period, so we regard this as the upper limit on the number of patterns available in a single experiment. Assuming parameter values available at the Linac Coherent Light Source (Emma *et al.*, 2010 ▸) (100 nm diameter beam and 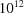 incident photons at 1.5 Å wavelength), we estimate that the correlation function from equation (8) for amorphous nickel could be measured with a signal-to-noise ratio (SNR) of 5 by collecting 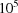 diffraction patterns, which requires less than 20 min of data collection at 100 Hz.

**Figure 4:**
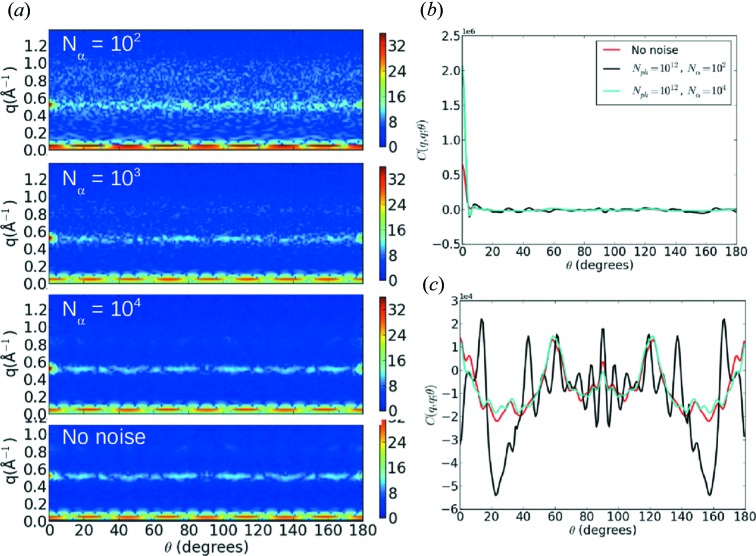
(*a*) The effect of noise on a cross-section of the intensity correlation function *C*(*q* = 0.5 Å^−1^, *q*′, θ) varying the number of patterns. Noise is clearly visible for 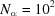, but is suppressed by 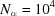 except for a large peak at 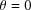 which arises from the self-correlation of the shot noise with itself. (*b*) A line plot of *q* = *q*′ = 0.5 Å^−1^ which shows that the peak at 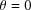 varies with the noise level. This peak can be removed with a centrosymmetric filter that replaces information in the vicinity of θ = 0° with the information near θ = 180°. (*c*) After applying the centrosymmetric filter, the 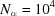 result agrees well with the noise-free simulation for the full range of θ.

**Figure 5:**
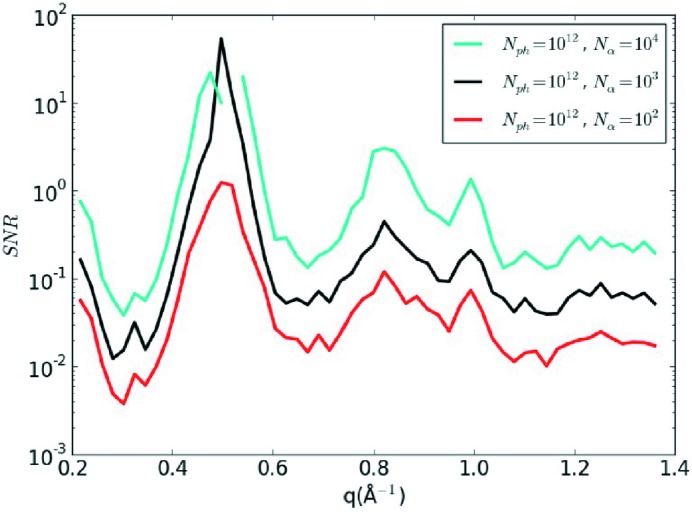
The numerically calculated SNR as function of the number of patterns. The signal is the standard deviation of the noise-free calculation and the noise level is calculated from the difference between the variance of each noisy simulation and that of the noise-free simulation. The increase of the SNR is proportional to *N*_α_^1/2^, as predicted by the theory in Appendix B[Sec appb].

We have verified these estimates for the required number of patterns with a numerical implementation of the statistical model. To avoid intensive MD simulations, we took the diffraction pattern calculated for a small sample volume (40 Å diameter; 2900 atoms), then scaled the mean scattering signal (as a function of *q*) by the number of atoms and scaled the fluctuations by the square root of the number of atoms, then combined both to create a new diffraction pattern. Shot noise was then calculated for the scaled diffraction pattern. The new pattern will have the correct ratio between shot noise and the interference terms between atoms with correlated positions. It does not model the noise from atoms with uncorrelated positions, which is a smaller effect than shot noise. To limit the number of MD simulations required, we randomly selected a sample state from 1000 different MD results and randomly rotated each selected structure. We fixed the experimental parameters at 100 nm diameter spot size, 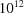 incident photons and 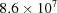 illuminated atoms. The number of atoms was calculated for a 100 nm sample thickness and a number density of 85.9 atoms nm^−3^ which was taken from the MD simulation. Fig. 4 ▸(*a*) shows cross-sections of 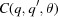 as 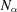 is varied. We see that the correlation function converges to the noise free calculation by 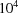 patterns, except for the self-correlation of the shot noise that generates a large peak when 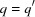 and θ = 0°, as shown in Fig. 4 ▸(*b*). This peak can be removed by applying centrosymmetry to replace the data near θ = 0° with the data measured around θ = 180°, which is appropriate if we ignore absorption. In our previous simulations centrosymmetry was already applied during the reconstruction of 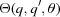. After filtering the noise peak, the 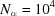 simulation agrees well with the noise-free simulation over the full range of θ as shown in Fig. 4 ▸(*c*). The numerically computed signal-to-noise estimates shown in Fig. 5 ▸ agree well with the analytic estimates, as the conservative limit of SNR = 5 has not yet been reached at most scattering angles for 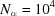.

**Figure 6:**
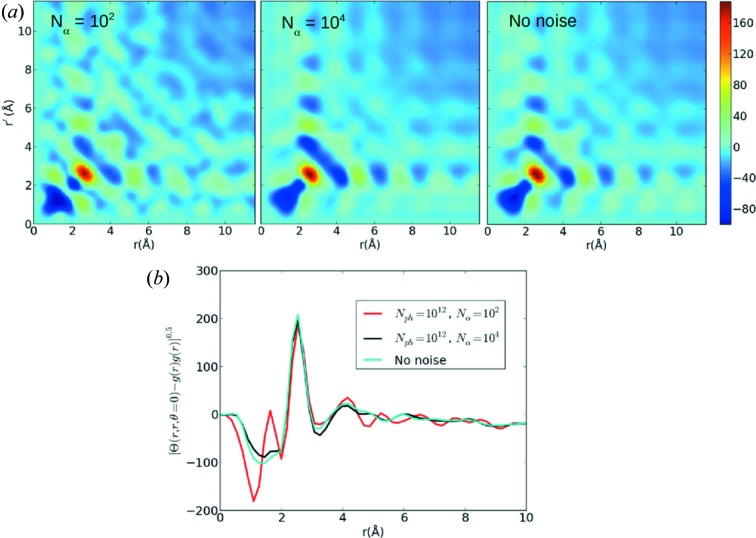
(*a*) The effect of noise on 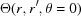 for amorphous nickel varying the number of patterns. The centrosymmetric filter has been applied to the intensity correlations. For 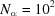, noise manifests in the reconstruction, but 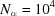 agrees well with the noise-free simulation. *b* The 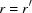 diagonal shows that 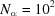 agrees with the noise-free simulation at the first-nearest-neighbour peak (2.5 Å), but disagrees elsewhere.

**Figure 7:**
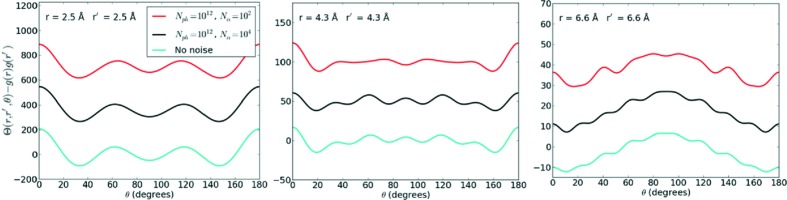
Angular distributions for amorphous nickel varying the number of patterns. The centrosymmetric filter has been applied to the intensity correlations. For 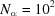, noise significantly the alters the peak structure beyond the first-nearest-neighbour distance (>2.5 Å). For 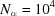, there is good agreement with the noise-free calculation. The plots have been offset to aid comparison.

Fig. 6 ▸ shows how the radial information contained in 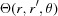 converges as the number of patterns increases. At 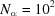 noise artefacts are apparent over the full range of *r* values, but disappear as the number of patterns increases to 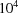, which shows good agreement with the noise-free simulation. Interestingly, even at 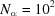 when shot noise exceeds the fluctuation signal for all scattering angles (the SNR of the intensity correlation is equal or below one), 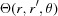 is still reasonably accurate in the strong first- and second-nearest-neighbour peaks as shown in Fig. 6 ▸(*b*). This may be because the extraction of 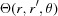 effectively applies an angular bandwidth limit and also because the calculation of the 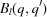 matrices is regularized. In other words, the diffraction fluctuations have a correlation on the detector that spans many pixels (*i.e.* oversampling) which are exploited in the conversion to real space to suppress noise. The angular distributions shown in Fig. 7 ▸ indicate that 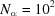 noise changes the angular peak structure of 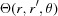 beyond the first-nearest neighbours, but these effects are largely gone by 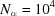.

Although we have taken indicative parameters from an XFEL for our noise analysis, it would be very interesting to explore whether these measurements can be made at nanofocus synchrotron beams, which are more accessible than XFELs. The number of X-rays available at a synchrotron source per second is comparable to the number in a single XFEL pulse. However, the efficiency of the X-ray focusing optics will be critical to delivering a high number of incident photons per measurement. For a continuous source, equation (20) shows that is it more advantageous to increase exposure time to reduce the number of measurements required. However, the maximum possible exposure time will be limited by other factors like radiation damage and instrument stability.

## 5. Conclusion   

We have shown that kinematic diffraction fluctuations can be mapped into a real-space correlation function that provides distributions of bond angles and orientational order. The correlations should be measurable with electrons for samples thin enough to avoid multiple scattering and with X-rays provided sufficiently high intensity and data rates can be achieved to overcome noise. Our analysis of the latter issue indicates that measurements of orientational order in metallic glasses, like nickel, should be within reach of current XFEL facilities and possibly nanofocus synchrotron beams by taking of the order of 10^4^–10^5^ measurements. Our noise model predicts that lighter elements such as carbon or oxygen will be be harder to measure and may require at least two orders of magnitude more measurements. The situation improves for organic molecules as then it is the number of molecules not the number of atoms that is relevant, as is known from the existing theory for the fluctuation diffraction of biomolecules.

There are a wide range of current applications for pair distribution analysis that can potentially be extended to the orientational analysis proposed here. Aside from glasses and amorphous solids, ultrafast X-ray or electron sources could be used to probe orientational order in liquid or gas phases (*e.g.* airborne particulate matter (Loh *et al.*, 2012 ▸)) because the ultrafast pulses outrun the translational and rotational motion of the sample. Ultrafast pulses could extend time-resolved small-angle or wide-angle X-ray scattering to orientational order. Real-space correlations could be used to add a new dimension to solution-scattering methods for biological structure, which were a key inspiration for our work. Further applications can be envisioned to heterogeneous biological systems such as unfolded or partially folded conformational ensembles (Lipfert & Doniach, 2007 ▸) and also to study the dynamics of these systems.

## Figures and Tables

**Figure 8:**
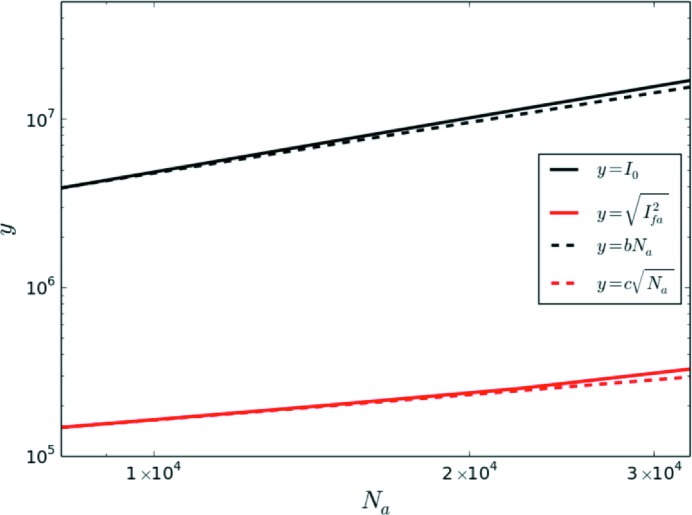
The scaling of 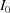 and 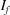 for nickel. The lines 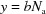 and 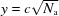 are given to indicate the scaling, where the parameters *b* and *c* are fixed so that the plots are equal to the simulations at 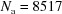 (corresponding to a radius of 30 Å).

## Appendix A Relationship between Θ(*r*, *r*′, θ) and correlation functions

In this appendix, we describe how  can be written in terms of correlation functions from statistical physics.

The *n*th-order atomic correlation function can be written as a formal count over the atoms in the sample using delta functions

where  is the position vector for atom ,  is the number of atoms contained in a sample volume *V*,  is the delta function and ρ is the number density (). The ensemble average is denoted by .

Kinematic scattering is sensitive to a reduced form of , because the absolute position of each atom pair is not measured, only the relative distance. We can express this by explicitly integrating out the unmeasured degrees of freedom. First we make a change of coordinates to place one atom at the origin (the reference atom), so that  and  is the relative displacement between the two atoms, which for clarity we denote . We then integrate out  as follows

To evaluate  we need to consider a two-body correlation function for a single instance of the sample  which has the same form as equation (21) but is not averaged over the ensemble. We then evaluate

Equation (23) resembles the fourth-order correlation function  with two reference atoms and two coordinates have been integrated out (the absolute position and the relative displacement between the two pairs), except that the limits of the sums over atoms include cases where atoms are identical, whereas equation (21) does not. In fact, the terms where two pairs of indices are equal (*e.g.*  and ) generate two-body correlations and terms where one pair of indices are equal generate three-body terms. Explicitly writing out the sums over identical atoms we find

where we have defined

and

We substitute this result into the expression for  and perform the angular integrations to obtain

where

and

where we have defined . The angular integrations can be performed by representing the delta functions in spherical coordinates. We choose relative coordinates for the second pair ( or ) such that the first atom pair  lies along the zenith direction. We then integrate over absolute orientation by integrating over the orientation of the first pair  and by integrating the relative azimuthal angle of the second pair . For example, the angular integration of a three-body correlation term, , is given by

## Appendix B Estimation of the required number of X-ray diffraction patterns

X-ray fluctuation diffraction has been demonstrated on 100 nm length scales, but to push to atomic length scales, we must understand how experimental parameters affect the size of the diffraction fluctuations and the feasibility of measuring them. Here we will use statistical arguments to determine the required number of patterns as a function of the other relevant experimental parameters. The arguments presented here are similar to those developed for the fluctuation diffraction of biomolecules (Kirian, 2012 ▸).

The intensity at a pixel can be modelled by

where  is the angular average of the intensity and  is fluctuation of the diffraction signal from the mean. The term  is a random variable that accounts for shot noise, which has a mean of zero. The standard deviation of  is equal to the square root of the intensity:

where  is the mean atomic structure factor and  depends on experimental parameters:

where  is the number of incident photons per measurement, *A* is the beam area,  is the classical electron radius and  is the solid angle of subtended by a pixel.

We assume that  can be measured accurately and subtracted prior to calculating the correlation function [equation (8)]. Evaluating an intensity correlation between a pixel at  and a pixel at  by summing over sample states α to obtain

The term  contains contributions from both correlated pairs, which we want to measure, and uncorrelated atomic pairs that are effectively an additional source of noise. Both  and  are additional sources of noise. It turns out that  is the dominant source of noise and to simplify this argument, this is the only source of noise we treat here. This is consistent with the analysis of Kirian *et al*. (2011 ▸) and we provide some further comment about other noise sources below.

We need to estimate the magnitude of the interference between correlated atom pairs and how it scales with the number of atoms. This was calculated directly from an MD simulation of amorphous nickel with  unit cells. For  Å, we used the simulated atomic structure to directly evaluate the diagonal terms of the matrices  (as defined in the *Methods* section) and estimated . We changed the number of atoms by selecting a spherical volume with a radius in the range 30–60 Å, which changed the number of atoms from 8517 up to 32457. Fig. 8 ▸ shows that  scales as the square root of the number of atoms *N*_a_^1/2^, while  scales with . Dividing  by  gives the contribution to  per atom [denoted ], which we can use to estimate the fluctuation signal for larger numbers of atoms than it is practical to simulate.

We then have that

where  is the standard deviation of the noise term. We note that  is correlated over an angular and radial range that is determined by the correlation length of the sample, whereas ∊ is uncorrelated between pixels. It is thus important to rebin the diffraction pattern before taking the correlation to maximize  and reduce noise. Denoting the number of pixels in the bin by , we find that  scales as , while  scales as . The correlation calculation also involves an integration over  angular bins that will further reduce noise. Finally the sum of the correlation over diffraction measurements  must be taken into account. When all of these operations are combined, we obtain the following expressions for the signal and the dominant error term:

We can then define a SNR, *R*, given by

which, after using equation (7) to replace , we rearrange in terms of the number of patterns,

Take the example of a  pixel detector positioned to measure 1 Å resolution at the detector edge. We choose an angular bin width of , so . The *q*-width of a pixel is around 100 nm, and we choose a radial bin of 40 pixels (sufficient to measure correlations between atom pairs up to 2.5 nm apart). Therefore, at 4 Å resolution which corresponds to 250 pixels distance from the beam center, a bin would contain close to 1560 pixels (). For our simulation of amorphous nickel,  and  at 4 Å resolution. We choose beam parameters with values achievable at the Linac Coherent Light Source (LCLS) (Emma *et al.*, 2010 ▸):  nm and . For a wavelength of 1.5 Å, the solid angle of a pixel is . To achieve  we find that , which to be conservative we round up to .

We note a few consequences of equation (40). The beam area *A* and the number of photons  are squared, which suggests that both of these parameters are critical for measuring the fluctuations in a feasible number of measurements. Interestingly equation (40) is independent of the number of atoms, which is because the noise and signal have the same dependence on this parameter. The number of patterns required is sensitive to elemental composition because . This means that lighter elements will be harder to measure, *i.e.* carbon would require around two orders of magnitude more measurements than nickel. The situation is more favourable for molecules, like proteins targeted by solution scattering methods, where  is proportional to the square of the scattering factor of the whole molecule, because all of the atoms within a rigid molecule are correlated.

We return now to make a few remarks about the noise generated by random correlations between local environments centred on different atoms. In the case of fluctuation diffraction of biomolecules the equivalent noise source is correlations between the diffraction of different molecules, and it well known that the signal-to-noise for this case is independent of the number of molecules and the beam intensity. It was suggested by Kirian *et al.* (2011 ▸) that this result may carry over to densely packed systems by taking a local arrangement of atoms as analogous to a molecule. Some care must be taken because in a densely packed system local structures are interconnected, whilst molecules are distinct objects. Nevertheless, this is confirmed by our simulations on volumes a few times the width of the sample’s structural correlation length. Larger sample volumes only increase the proportion of distinct local structures for which the assumptions of the biomolecule noise analysis are valid.

Our analysis has assumed a uniform beam profile, which is partially justified for X-ray beams because the spatial variation in the beam is typically on larger length scales that the correlation length of amorphous materials (>2 nm). The overall intensity distribution can affect our signal-to-noise analysis. For example, some X-ray beams have broad ‘tails’ that, although weak, can spread over a large area and account for a significant fraction of the total photons. Thus, an extension of our noise analysis to non-uniform beam profiles will be very important for X-ray experiments and forms part of ongoing work. Our current understanding is that the signal-to-noise varies with the ratio of the variance of the beam intensity to the mean beam intensity, which means the bright focus of the beam is expected to make a much greater contribution to intensity correlation measurements than the beam tails.

Finally, we note that rebinning the data to an effective pixel size induces an effective traverse coherence length. When matched to the structural coherence length of the sample it effectively suppresses the noise from the interference between uncorrelated atoms larger than this length in addition to shot noise. Essentially, the interference from uncorrelated atoms produces fringes much finer than a pixel width, which have close to zero contribution to the integrated pixel intensity.
